# Poorly differentiated neuroendocrine carcinoma originating in the subglottic larynx: A case report with imm unohistochemical study

**DOI:** 10.1002/ccr3.8792

**Published:** 2024-04-23

**Authors:** Mahboobe Asadi, Milad Elyasi, Arvin Shahzamani, Saeedeh Miri

**Affiliations:** ^1^ Otolaryngology Department Shahid Beheshti University of Medical Sciences Tehran Iran; ^2^ Pathology department Tehran University of medical sciences Tehran Iran

**Keywords:** laryngeal carcinoma, neuroendocrine carcinoma, pathology, subglottic larynx

## Abstract

**Key Clinical Message:**

Laryngeal neuroendocrine carcinomas are the most common non‐squamous neoplasm of the larynx. Due to the rarity of the tumor, pathological diagnosis should be confirmed by immunohistochemistry.

**Abstract:**

Laryngeal neuroendocrine carcinomas (LNECs) are a rare cancer of the head and neck. Few case reports of poorly differentiated neuroendocrine carcinoma originating in the subglottic larynx exist within the literature. In this case, we discuss a 57‐year‐old patient with a history of four‐month hoarseness with a newly diagnosed of poorly differentiated neuroendocrine carcinoma in the subglottic larynx. Treatment and prognosis of the various NEC groups differ, so precise identification requires consideration of the microscopic findings and immunostaining analysis. immunohistochemistry staining demonstrated positive result for cytokeratin 7, synaptophysin, chromogranin, CD 56, with the Ki‐67 index of45%. Although surgery is usually the treatment for all tumor types, chemo radiotherapy is recommended for poorly differentiated NECs because surgery is ineffective.

## Graphical abstract

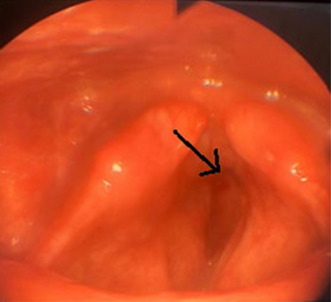

## 1 INTRODUCTION

Laryngeal neuroendocrine (NE) neoplasms are rare tumors of the larynx with a wide spectrum of clinicopathological manifestations that exhibit NE differentiation. Laryngeal NENs are extremely rare, comprising <1% of all laryngeal neoplasms. Generally, NE neoplasms are categorized into three groups based on histology: Well‐differentiated NE tumors (grade 1–3), neuroendocrine carcinoma (NEC) (Small cell NEC and large cell NEC) and Mixed NE non‐NE neoplasms.^1^ The first case of laryngeal NENs of the larynx was reported by Goldman et al in 1969. To date more than 700 cases of LNENs have been reported in the literatures.^2^ Laryngeal NE neoplasm occurring more frequently in men in their fifth to seventh decade of life. The supraglottic region is the most frequent location.^3^

Although, clinical symptoms are usually nonspecific and related to obstructive mass lesion, in rare cases, paraneoplastic syndrome present due to hormone overproduction by the tumor.^4^ Besides, laryngeal NENs commonly show NE immunohistochemical features, including expression of chromogranin A (CGA), synaptophysin (SYP), and cytokeratin. Interestingly, some cases of NECs may exhibit negative stains for some first‐generation NE markers. In those patients second‐generation markers such as INSM transcriptional repressor 1 (INSM1), ISL LIM homeobox 1 (ISL1), and secretagogin (SECG)could be valuable.^5^

Clinical diagnosis and prognosis of laryngeal NENs differ widely from squamous cell carcinomas (SCC). Based on the tumor rarity and wide spectrum of pathological features, an accurate diagnosis is of paramount importance.

In this study, we presented a rare case of poorly differentiated NEC (small cell) and described its clinicopathological presentation. The presentation of this tumor with respiratory distress in a nonsmoker male patient with a small size mass in subglottic area is the interesting feature of this report.

## 2 CASE HISTORY

**FIGURE 1:**
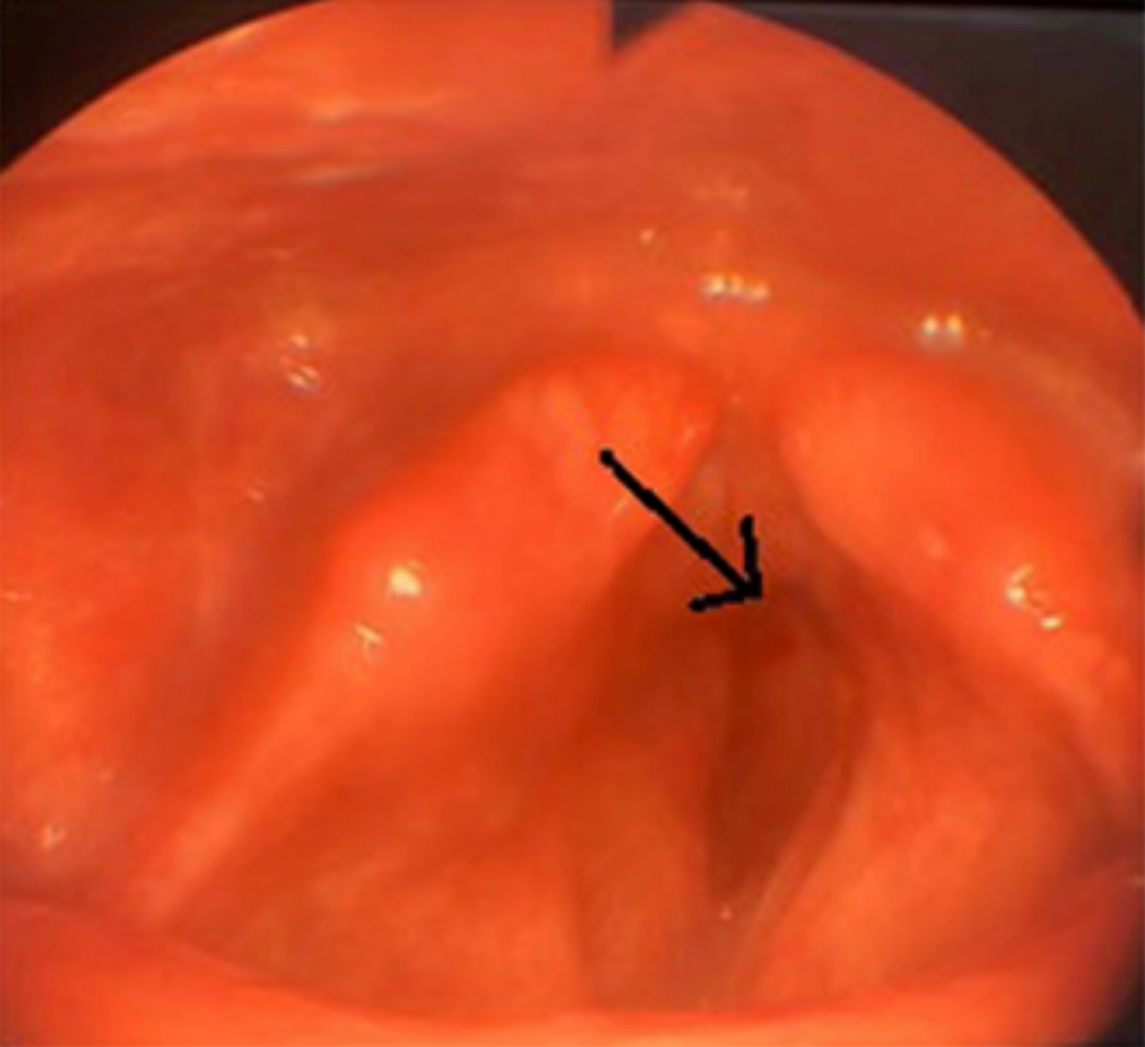
Laryngoscopic images showing a red mass in the sub glottis (black arrow).

A 57‐year‐old nonsmoker man referred to our otolaryngology clinic with a history of four‐month hoarseness. He also complained of progressive dyspnea without cough or aspiration. Direct laryngoscopy revealed a smooth non‐ulcerated tumor in the sub glottis and vocal cord fixation in the right side (Figure 1). The mentioned lesion had a red appearance without necrosis. On neck examination, an enlarged cervical lymph node was palpated in level 6. His general condition was good.

## 3 METHODS

**FIGURE 2:**
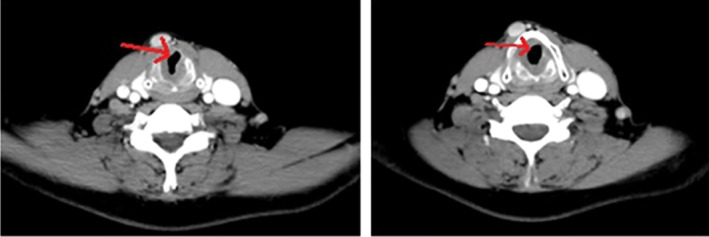
Contrast‐enhanced computed tomography scan showing subglottic tumor (red arrow).

The computerized tomography scan showed a small lesion in the subglottic with the evidence of right true vocal cord fixation. Supraglottic was free of tumor. Also, an enhanced metastatic cervical lymph node was detected in the pre laryngeal area (Figure 2). Moreover, on the chest, abdominal computerized tomography scan, and bone scan no distant metastatic lesion was noted.

**FIGURE 3:**
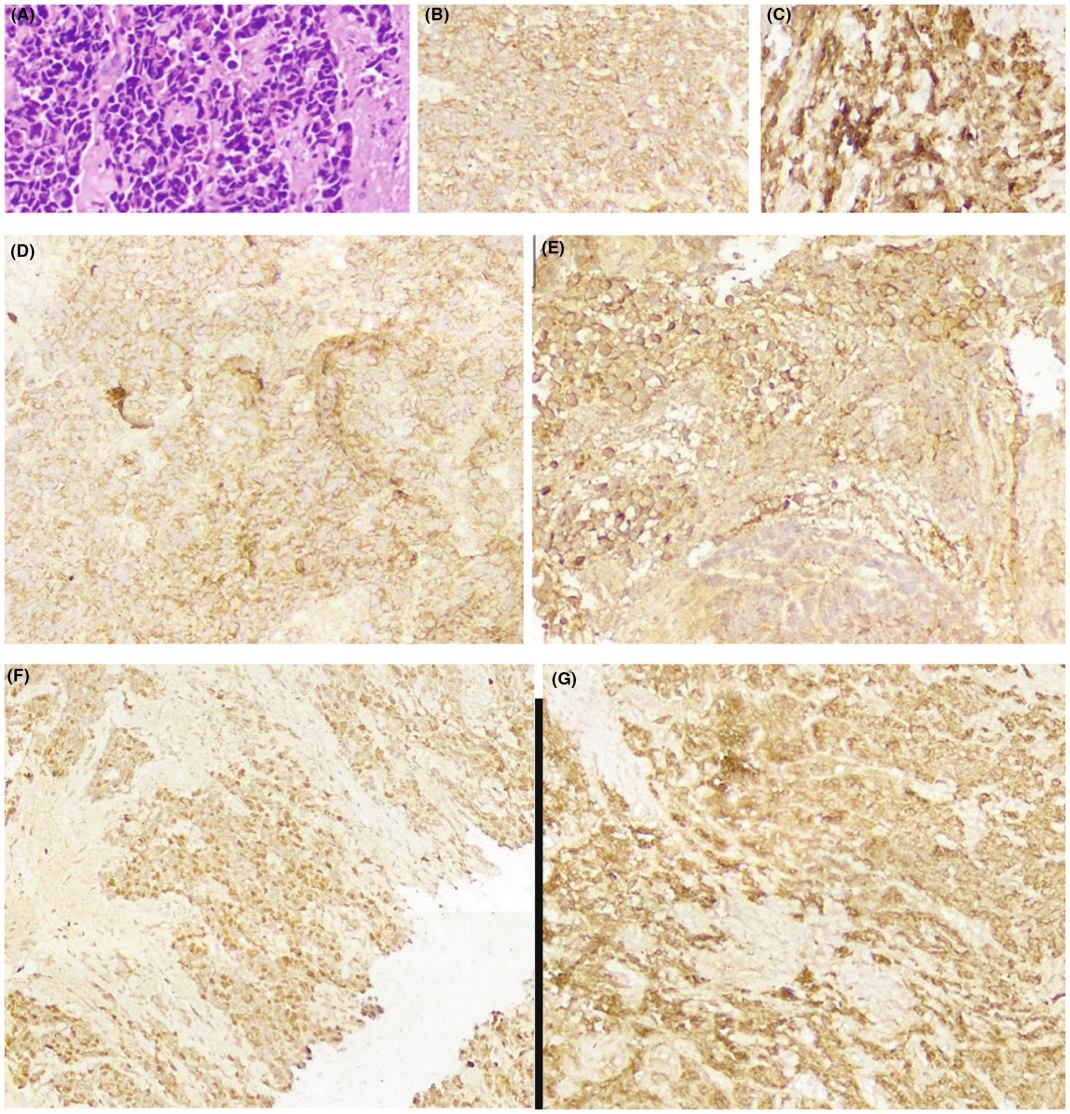
Overview of pathology sections. (A–G) H&E stain, Synaptophysin IHC, Chromogranin IHC, CK IHCstain, CD56 IHC stain, KI 67 index, cytokeratin 7 IHC stain.

Direct laryngoscopy and biopsy were performed under general anesthesia. The histopathology findings were suggestive for NEC. Microscopic histopathology analysis showed a tumor consist of small size neoplastic cells with acidophilic cytoplasm, coarse chromatin pattern with areas of necrosis, and a high mitotic rate (more than 10/mm^2^). Immunohistochemistry was positive forcytokeratin7, SYP, chromogranin, cluster of differentiation (CD) 56, with Ki67 index of 45% (Figure 3). These findings were consistent with the diagnosis of small cell NEC.

Our case presented as a localized NEC involved subglottic larynx, but right vocal cord fixation represents the tumor extension to glottic region. Based on this evidence it was not a real localized tumor. Since chemo radiotherapy is recommended for poorly differentiated NECs, the patient was referred to the radiation oncology department.

## 4 CONCLUSION AND RESULTS

After 2 weeks of therapy, the patient developed respiratory distress, characterized by biphasic stridor, which required tracheotomy. After 4 months from the beginning of the therapy, the sub glottis mass lesion, and the cervical lymph node disappeared. Then, the tracheostomy tube was removed, and the patient was advised to follow up.

## 5 DISCUSSION

We describe a rare case of laryngeal NEN in subglottic. While many laryngeal NEC found in the supraglottic, in about 60%–96% of casese.^6^

Although primary NEC are rare, they are the most common non‐squamous neoplasm of the larynx. NE neoplasms are categorized into three groups based on histology: Well‐differentiated NE tumors (grade 1–3), NEC (Small cell NEC, and large cell NEC), and Mixed NE non‐NE neoplasms.^1^ Moreover, based on NEC classification as suggested by the International Agency for Research on Cancer (IARC) and World Health Organization (WHO), in poorly differentiated epithelial neoplasms, Microscopic histopathologic analysis showing tumor necrosis, and mitotic rate of >10/2 mm^2^.Small cell NEC of larynx are immunoreactive for AE1/AE3 and CAM5.2. Although, high‐molecular‐weight cytokeratin (CK5/6 and CK903) and p63 are commonly negative, it may be focally positive in some cases. Moreover, markers such as SYP, CD56, and neuron specific enolase, are positive.^7^

Besides, small cell NECs of various sites may exhibit negative stains for some first‐generation NE markers such as chromogranin A (CGA) and SYP and those second‐generation markers such as INSM transcriptional repressor 1 (INSM1), ISL LIM homeobox 1 (ISL1), and secretagogin (SECG) could be valuable in this aspect.TTF‐1 may show nuclear staining in some cases.^5^ TTF1 and calcitonin exhibited negative stains in our patient.

Furthermore, in a study of 229 Pan NE neoplasms NENs, calcitonin expression was detected in 10.9% of the cases. Moreover, calcitonin can be a NE marker of laryngeal NENs, only six cases of laryngeal NENs with elevated serum calcitonin levels were described in the literature. However, in our case calcitonin expression was negative and serum calcitonin level was normal.^8^

Atypical carcinoid is the most common NECs of the larynx that represents 0.2%–0.6% of laryngeal malignancies.^3^ Also, atypical carcinoid has more aggressive character that metastasis to cervical lymph nodes, bone, skin, liver, and lung are frequent in their disease nature.^9^, ^10^ Laryngeal NENs typically affect middle‐aged male patients with a history of heavy smoking.^11^ The common clinical manifestations are Hoarseness, dysphagia, and sore throat. In rare cases paraneoplastic syndrome happens. Moreover, cervical lymph node metastases and cutaneous metastases may be detected in some cases.^8^ Despite the small tumor size in our patient, cervical lymph node metastasis was detected.

Treatment and prognosis of the various NEC groups differ; so precise identification of tumor group is critical. Although surgery is usually the treatment for all tumor types, chemotherapy may be utilized for moderately to poor differentiated NECs. Laryngeal well‐differentiated NEC is treated with wide local excision, generally a partial laryngectomy, without neck dissection. While, in moderately differentiated NECs partial or total laryngectomy with elective or therapeutic neck dissection is necessary. However, adjuvant chemo radiotherapy may be beneficial in some cases. Eventually, chemo radiotherapy is recommended for poorly differentiated NECs because surgery is ineffective in these groups.^12^ the right vocal cord fixation in our case demonstrated the tumor extension from subglottic to glottic. Based on tumor extension and the nature of poorly differentiated NECs, our patient was referred to radiation oncology department. Furthermore, tumor prognosis differs among various tumor types. A 5‐year disease‐specific survival rate is about100%, 53%, 19%, and 15%, in Well‐differentiated NEC, moderately differentiated NEC, small cell NEC, and large cell NEC respectively.^6^

In conclusion, the differential diagnosis in a patient with hoarseness, dyspnea, and subglottic mass must consist of NEC. Due to tumor rarity, pathological diagnosis should be confirmed by immunohistochemistry. Irradiation and chemotherapy are recommended for treatments in poorly differentiated NEC. This case report of poor differentiated NEC and the corresponding literature review provide useful insight for otolaryngologists to improve their knowledge in the diagnosis and treatment of this aggressive neoplasm.

## AUTHOR CONTRIBUTIONS

**Mahboobe Asadi:** Conceptualization; data curation; formal analysis; investigation; methodology; project administration; resources; software; supervision; validation; visualization; writing – original draft; writing – review and editing. **Milad Elyasi:** Funding acquisition; visualization; writing – original draft; writing – review and editing. **Arvin Shahzamani:** Investigation; supervision; validation; visualization; writing – original draft; writing – review and editing. **Saeedeh Miri:** Data curation; formal analysis; resources; writing – original draft; writing – review and editing.

## FUNDING INFORMATION

None.

## CONFLICT OF INTEREST STATEMENT

The authors made no disclosures.

## ETHICS STATEMENT

Because this report involves no experiment, ethics approval is waived.

## CONSENT

Written informed consent was obtained from the patient for publication of this case report and accompanying images. A copy of the written consent is available for review by the Editor‐in‐Chief of this journal on request.

## Data Availability

The data that support the findings of this study are available on request from the corresponding author, [Mahboobe Asadi].
